# Expression of TNF-*α* and IL-1*β* in Peripheral Blood of Patients with T2DM Retinopathy

**DOI:** 10.1155/2022/9073372

**Published:** 2022-08-08

**Authors:** Jin Qian, Ying Huang

**Affiliations:** Medical Laboratory Center, Jiangsu Taizhou People's Hospital, Taizhou, 225300 Jiangsu Province, China

## Abstract

**Aims:**

The expression and clinical significance of tumor necrosis factor-*α* (INF-*α*) and interleukin 1-*β* (IL-1*β*) in retinal cells of patients with type 2 diabetes (T2DM) retinopathy were detected by flow cytometry.

**Materials and Methods:**

Fifty patients with T2DM who attended our ophthalmology clinic between May 2021 and May 2022 were selected as the observation group. Another 50 healthy individuals who were examined at our hospital during the same period were selected as the comparison group. Tear film rupture time (BUT), fluorescein staining (FL), basal tear secretion (Schirmer I) test, and conjunctival impression cytology (CIC) were detected in both groups, and the expression of TNF-*α* and IL-1*β* in retinal cells was observed by immunohistochemical staining.

**Results:**

The levels of IL13 and TNF-*α* in the two groups were not exactly the same. The serum levels of IL13 and TNF-*α* in the observation group were significantly higher than those in the control group, and there was a statistically significant difference (*P* < 0.05). TNF-*α* and IL-1B expressions in the observation group were positively correlated with the fluorescence staining, and the expression of TNF-*α* and IL-1*β* in the observation group was significantly negatively correlated with the BUT test and Schirmer I test.

**Conclusion:**

Serums TNF-*α* and IL-1*β* are significantly elevated in patients with T2DM retinopathy and gradually increase with disease progression. Combined detection of serums TNF-*α* and IL-1*β* can help determine the severity of the disease and assess the prognosis.

## 1. Introduction

Diabetic patients often complain of dryness, burning, and foreign body sensations in the eye and other symptoms such as reduced corneal perception and edema. This brings inconvenience to the life of diabetic patients, so diabetic ocular surface disease has attracted great clinical attention [1]. Recent experimental studies in foreign animal models have reported that the P38 mitogen-activated protein kinase (MAPK) signaling pathway is mainly involved in the inflammatory response under stress, and a variety of inflammatory stockiness and some stress responses can activate the signaling pathway and regulate many inflammatory expressions, for which the inflammatory factor tumor necrosis factor-*α* (INF-*α*) and interleukin 1-*β* (IL-1*β*) are key factors. Another important signaling pathway is phosphatidylinositide 3-kinase (PI3K)/protein kinase B (Akt), which plays a role in cell growth, proliferation, proliferation, and inflammation. Another important instruction pathway is PI3K/Akt, which plays an important role in cell growth, proliferation, survival, and apotheosis [2]. Therefore, by interfering with the expression of key signaling molecules in the P38/MAPK and PI3K/Akt signaling pathways in clinical treatment, it is possible to block the signaling pathways of their associated inflammatory pathways and block the inflammatory response [3]. Retinopathy in type 2 diabetes (T2DM) is a common complication of T2DM, and the main pathological manifestations are retinal microvascular damage, basement membrane thickening, and regularization [4]. Both hyperglycemia and long-term stimulation of late coagulation end products (Age) can lead to local aerodynamic abnormalities of the eye, leading to hypoxia of retinal tissue, vascular epithelial damage and inflammatory cytosine activation, disruption of the blood retinal barrier, and ultimately diabetic retinopathy (DR) [5].

In the inflammatory loop of T2DM homoeopathy, two factors, TNF-*α* and IL-1*β*, also occupy an important position, among which TNF-*α* tumor necrosis factor, which functions to cause tumor chemise necrosis and tumor cell death, has a wide range of biological activity [6]. Epithelial and epithelial cells activated by TNF-*α* can produce IL-1*β*, which can act as an immune mediator in inflammation development [7]. This shows that the two factors play complementary roles in the inflammatory loop of T2DM homoeopathy and together promote the formation of ocular surface inflammation [8]. TNF-*α* and IL-1*β* are two factors with inflammatory significance in the P38MAPK and PI3K/Akt signaling pathway in T2DM patients with induced T2DM homoeopathy, which can reflect the inflammatory state of the ocular surface [9]. Our study analyzed the expression of TNF-*α* and IL-1*β* in the peripheral blood of patients with T2DM homoeopathy, aiming to provide more references for the clinical treatment of patients.

## 2. Material and Methods

### 2.1. Research Object

Fifty patients with T2DM who attended our ophthalmology clinic between May 2021 and May 2022 were selected as the observation group, and 50 healthy people who underwent physical examination in our hospital during the same period were selected as the comparison group. The diagnosis of T2DM was based on the diagnostic criteria of T2DM established by WHO [10] (fasting blood glucose greater than or equal to 7.0 mole/L, or two-hour postprandial blood glucose greater than or equal to 11.1 mole/L); T2DM homoeopathy conforms to the international clinical DR severity grading criteria in 2002 [11], i.e., the patient's fundus shows characteristic retinal micromanage, hemorrhages, hard extenuates, soft extenuates, oracular edema, retinal regularization, etc. DR proliferation stage: regularization appears on the retinal surface or in front of the retina, or there is vitreous hemorrhage, fiber proliferation, and retinal detachment. Patients in the healthy comparison group were required to have no disorders of Coolidge metabolism, no family history of T2DM, and no abnormalities in fundus examination.

### 2.2. Inclusion and Exclusion Criteria

Inclusion criteria are as follows: observation group: (1) the selected patients were in the nonproliferation stage of DR; (2) the history of T2DM reached more than 8 years and had good hypoglycemic control; (3) there was a clear history of type 2 T2DM, meeting the diagnostic criteria of T2DM homoeopathy, and the diagnosis of nonproliferation stage by fundus fluorescence bibliography, aged from 40 to 80 years old, both sexes were eligible. Comparison group: no ocular medication, no corneal contact lens, no ocular laser surgery, no ocular surface-related ocular trauma, no ocular-related surgery, no systemic diseases, and no other ocular diseases within the last three weeks.

Exclusion criteria are as follows: (1) pregnancy, air breast women, abnormal liver function, T2DM telepathy kidney failure (toxaemia phase, uremic phase), history of allergies, unsuitable for fluoroscope, psychiatric patients, combined with cardiovascular, liver, kidney, and hematologist system and other serious primary diseases; (2) other eye diseases combined (such as glaucoma, cataracts that significantly affect the fundus examination, non-T2DM Homoeopathy, and ileitis); (3) patients with other eye diseases (such as glaucoma, cataract that significantly affects fundus examination, non-T2DM homoeopathy, and ileitis), those who have worn corneal contact lenses, laser eye surgery, ocular surface-related eye trauma, and ocular-related surgery; and (4) patients with T2DM, T2DM during pregnancy, and other special types of T2DM, combined with connective tissue disease or other autoimmune diseases, those who have used hormones and noninflammatory drugs within the last 3 months, combined with severe liver and kidney insufficiency, malignant tumors, or acute and chronic patients with acute and chronic infections.

## 3. Methods

Tear film rupture time measurement (BUT): use sterile forceps to take saline-soaked and then lightly touch the florescent sodium test paper of the patient's lower lid instructing the patient to blink sometimes, lightly touch the patient's lower face conjunct sac, instruct the patient to blink a number of times, and then look flatly ahead, live microscope cobalt blue light under wide slit light band observation, stopwatch timing, open the eyes after the last blink until the first black spot appears in the cornea, and record the average value after 3 times when the BUT value < 10 s was considered abnormal. Florescent staining (FL): aseptic forceps were used to take sodium fluorescent test paper after wetting with physiological saline, lightly touching the patient's lower lid conjunct capsule, asking the patient to blink several times, and then looking flatly ahead, cobalt in vivo microscopy, and wide slit light band observation under blue light. Scoring: the cornea was divided into 4 quadrants, grade 0: no staining (-), grade 1: scattered dot staining (+), grade 2: dense dot staining (++), and grade 3: Bellamy staining (++++). The scores 3, 2, 1, and 0 were recorded, respectively.

Basic tear secretion test: without surface anesthetic, reverse-fold the tear detection filter paper strip at the 0-point blank and gently place it in the conjunct membrane of the lower third of the outer and outer eyelid, put it down and ask the patient to gently close the eyes to avoid stimulating excessive tear production, remove the tear detection filter paper strip 5 minutes after starting the timing, and read the wetting (discoloration) length of the filter paper from the reverse-folded 0-point, and the wetting length < 10 m is abnormal.

Blot cytology: 3 mm × 3 mm square nitrocellulose filter paper was disinfected by UV light irradiation before the experiment. Anesthesia: procaine hydrochloride binocular solution was dabbed in the right eye of both groups of patients, and surface anesthesia was performed for about 5 min, and any excess tears or anesthetics were removed by aspiration with the filter paper. Collection and fixation: the edges of the nitrocellulose filter paper sheets were gently clamped with flat forceps and pressed firmly against the ha side of the conjunct surface of the right eye, and the filter paper was removed after 10 seconds of gentle pressure and fixed in 95% ethanol to correspond to the markings. Staining: periodic acid-Schiff (PAS) staining was applied. Gradient alcohol was dehydrated, ethylene was transparent, and the number and status of scapular cells were observed and recorded under light microscope at 400x.

Photochemical staining: nitrocellulose films were cut into 3 mm × 3 mm squares and sterilized for use. Antidepletion slides were prepared. In both groups, procaine hydro chloride eye solution was spotted in the right eye, and surface anesthesia was performed for about 5 min, and any excess tears or anesthetics were removed using filter paper. The edge of the nitrocellulose filter paper sheet should be gently clamped by a flat regent and pressed tightly against the right eye conjunct surface after the title side, and the filter paper was removed after 10 seconds of gentle pressure, and the specimen was fixed on the slide and marked. The nitrocellulose film blot slides were fixed in 95% ethanol. Anhydrous alcohol was dehydrated and then rinsed with water for microwave repair. 0.3% H2O2 was incubated for 10 min at room temperature to drive off the endogenous peroxide enzyme reaction. Distilled water rinsing is followed by 0.01% PBS drops washing for 5 min three times. Add a nonstaining blocking solution for 60 min at room temperature to remove nonspecific staining, add monoclonal antibody and incubate overnight at 4°C, and rinse with PBS for 5 min for a total of three times. Add horseradish-labeled secondary antibody and place in a 37 degree Celsius incubator for 1 hour, and rinse with PBS for 5 minutes 3 times. DAB horseradish hydroxide staining: 0.5 mL each of DAB staining solutions A and B, 1 dab staining working solution (ration at any time of the experiment) at room temperature and protected from light for 30 in. Distilled water wash for 5 minutes to terminate the reaction. Bayer hematology staining for 2 minutes, rinse with tap water for 10 minutes. Specimens were rinsed with tap water for 10 min. The blotted pieces were fixed in 95% alcohol, dried in anhydrous alcohol, and homogeneously transparent in ethylene, and the blotted pieces were sealed with treacle. The following criteria judged IL-1B and TNF-*α* staining results: observed by light microscope, clear cytologist boundary, no cytoplasmic staining was recorded as negative (-); light cytoplasmic staining, showing light brown color was recorded as weakly positive (+); and moderate cytoplasmic staining, showing brown color was recorded as moderate positive (++). Significant cytoplasmic staining with a dark brown color was recorded as a strong positive (++++).

### 3.1. Statistical Methods

All data of our study were checked using Excel double entry and SPSS 28.0 for statistical analysis, setting the test level *α* = 0.05 and considering *P* < 0.05 as a statistically significant difference. Statistical descriptions of measurement data that obeyed normal distribution were described by mean ± standard deviation, and those not obeying normal distribution were described by median (interquartile spacing), and count data were described by frequency and composition ratio. General patient data were analyzed: categorical data were analyzed by chi-square test, continuity-corrected chi-square test, and Fisher's exact probability method; measurement data were analyzed by *t*-test. Obedience to a normal distribution, paired-sample *t*-test was used for within-group comparisons and two-independent sample *t*-test for between-group comparisons; disobedience to normal distribution, nonparametric constellation signed-rank test was used for within-group comparisons, and rank-sum test was used for between-group comparisons for analysis.

## 4. Results

### 4.1. Baseline Data Comparison

The mean age, gender, body mass index, and weight of the patients in the observation group were not statistically significant compared with the comparison group (*P* >0.05) (see Table 1).

### 4.2. Comparison of IL-1*β* and TNF-*α* Levels

**Figure 1:**
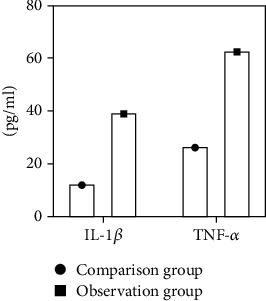
Comparison of IL-1*β* and TNF-*α* levels. In our study, all data were checked by double entry in Excel, and SPSS 28.0 was used for statistical analysis. The statistical descriptive measurement data obeyed the normal distribution. They were described by the mean ± standard deviation. Paired sample *t*-test was used for infragroup comparison, and two-independent sample *t*-test was used for intergroup comparison. It was found that a one-way ANOVA comparison of the two groups showed that the levels of IL-1*β* and TNF-*α* were not identical between the two groups. Further comparison showed that the serum IL-1*β* and TNF-*α* levels of patients in the observation group were significantly higher than those in the comparison group, with statistically significant differences (*P* < 0.05), ^∗^*P* < 0.05.

A one-way ANOVA comparison of the two groups showed that the levels of IL-1*β* and TNF-*α* were not identical between the two groups. Further comparison showed that the serum IL-1*β* and TNF-*α* levels of patients in the observation group were significantly higher than those in the comparison group, with statistically significant differences (*P* < 0.05) (see Figure 1).

### 4.3. The Relationship between TNF-*α* and IL-1*β* Cytokines

**Figure 2:**
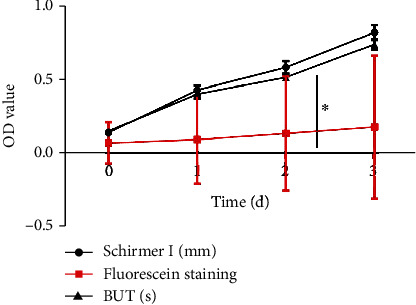
The relationship between TNF-*α* and IL-1*β* stockiness. In our study, all data were checked by double entry in Excel, and SPSS 28.0 was used for statistical analysis. The statistical descriptive measurement data obeyed the normal distribution. The mean ± standard deviation described them. Paired sample *t*-test was used for infragroup comparison, and two-independent sample *t*-test was used for intergroup comparison. It was found that the expressions of TNF-*α* and IL-1*β* in the observation group were positively correlated with fluorescence staining, and the expressions of TNF-*α* and IL-1*β* in the observation group were significantly negatively correlated with the BUT test and Schemer I test. The results indicated that fluorescent staining, BUT, and Schemer I experiments reflected the patient's progress.

The expressions of TNF-*α* and IL-1*β* in the observation group were positively correlated with fluorescence staining, and the expressions of TNF-*α* and IL-1*β* in the observation group were significantly negatively correlated with the BUT test and Schemer I test. The results indicated that fluorescent staining, BUT, and Schemer I experiments reflected the patient's progress (see Figure 2).

### 4.4. Comparison of the Expression of TNF-*α* and IL-1*β* in Retinal Cells

**Figure 3:**
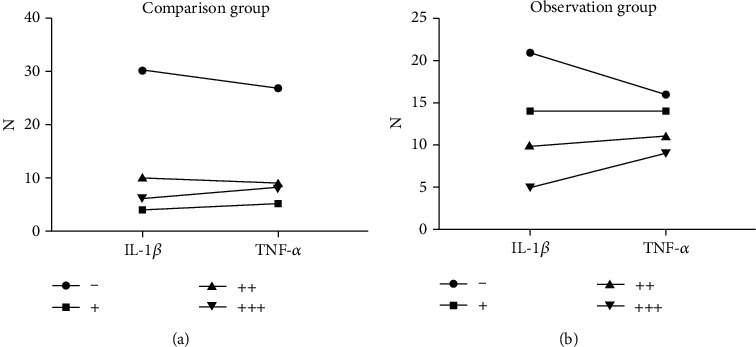
Comparison of the expression of TNF-*α* and IL-1*β* in retinal cells. In our study, all data were checked by a double entry in Excel, and SPSS 28.0 was used for statistical analysis. The statistical descriptive measurement data obeyed the normal distribution. The mean ± standard deviation described them. Paired sample *t*-test was used for infragroup comparison, and two-independent sample *t*-test was used for intergroup comparison. It was found that comparing the expression rates of stockiness between the two groups, the expressions of TNF-*α* and IL-1*β* in retinal cells in the observation group were significantly increased compared with those in the control group, with a statistically significant difference (*P* < 0.05).

Comparing the expression rates of stockiness between the two groups, the expressions of TNF-*α* and IL-1*β* in retinal cells in the observation group were significantly increased compared with those in the control group, with a statistically significant difference (*P* < 0.05) (see Figure 3).

## 5. Discussion

The results of early animal model studies of T2DM showed a large number of white blood cell aggregates in retinal macular cells and a significant increase in several inflammatory factors, including interleaves and TNF-*α* [12]. A comparative study of T2DM patients undergoing hysterectomy found that IL-1*β* and TNF-*α* were significantly elevated in vitreous bodies without proliferative homeopathy, confirming the role of inflammatory factors in developing DR [13]. There are two types of IL-1, IL-1*α*, and IL-1*β*, and IL-1 in the blood is mainly IL-1*β* secreted by monocycle and esophageal. IL-1*β* binds to its receptor and is involved in the inflammatory response through G protein coupling for signal instruction [14]. IL-1*β* can cause intramural infiltration and diffusion of serum proteins and erythrocytes, leading to local exudation, edema, inflammation, widening of the vascular epithelial space, and destruction of the blood retinal barrier [15]. IL-1*β* can regulate immune cells and induce epithelial cells to express cell-free adhesion factors, is a strong chemotactic agent that enhances nutrition, and plays an important role in leukocyte adhesion and activation [16]. In addition, IL-1*β* is involved in the formation of nonproliferation homoeopathy by acting on retinal pigment epithelial cells and promoting collagen synthesis and deposition [17]. One study found that serum IL-1*β* was elevated in patients with T2DM homoeopathy compared to the comparison group, suggesting that elevated IL-1*β* levels are an important factor in DR. TNF-*α* is mainly secreted by denuclearize esophageal and is an inflammatory cytosine with multiple biological functions [18]. First, TNF-*α* is involved in developing insulin resistance and promotes the progression of T2DM and its complications [19]. TNF-*α* induces activation of the NFL pathway by acting on receptors on vascular epithelial cells, causing the appearance of retinal vascular cells, increasing vascular permeability, stimulating the proliferation of additional vascular stromal and vascular cells, and promoting binocular normalization [20]. TNF-*α* can also increase the activity of eyeglasses and promote abnormal expression of adhesion factors, which enhances the local adhesion and aggregation of inflammatory cells and releases radioactive mediators in the retina, stimulating the local inflammatory response to form a vicious circle [21]. Studies have shown that TNF-*α* is significantly correlated with the severity of the disease, and that inhibition of TNF-*α* expression reduces retinal vascular epithelial cell apotheosis by about 80% [22].

It is currently believed that the P38MAPK and PI3K/Akt instruction system triggers inflammatory responses by enhancing TNF-*α* expression to promote cellular regulation. Conversely, TNF-*α* factors can act as triggering factors to stimulate signaling pathways [23]. In recent research trials, signaling pathway inhibitory drugs have become a hot issue in treating chronological and inflammatory diseases, with little relevant research on T2DM homoeopathy [24]. Our experiment indirectly responded to the activation level of the P38MAPK and PI3K/Akt instruction system by detecting TNF-*α* and IL-1*β* stockiness, formulators of signaling pathways, to provide a basis for subsequent therapeutic studies in ocular surface diseases [25]. TNF-*α* has a wide range of biological activities, and crucially, it can also direct the production of IL-1*β* by inflammatory cells [26]. The detection factor IL-1*β* in our experiments is precursor, which has similar biological activities, multifunctional biological roles, and potent inducers of TNF-*α* [27].

We used a photochemical staining assay to detect the levels of TNF-*α* and IL-1*β* production and thus analyze the inflammatory mechanisms of T2DM homoeopathy caused by T2DM patients. The results showed that when the level of inflammation of the retinal epithelium was higher than normal, the ocular surface condition of patients with T2DM was in a state of increased inflammatory activity, which may lead to abnormal changes in the normal tear film on the surface of the eye, thus aggravating the progression of homeopathy in T2DM. The results of related studies have shown that impregnation of TNF-*α* and IL-1*β* in the retina affects the normal release of transmitters in transmissible [28]. To study the epidemiological changes in the ocular surface of mice with T2DM homoeopathy, a model of increased production of inflammatory factors TNF-*α* and IL-1*β* in the conjunctiva of mice was made by inhibiting the general function of choleric receptors in the lackey gland. The results showed that this model could cause a decrease in tear secretion [29]. In contrast, it was found that rat corneal epithelial cell lines produce inflammatory stockiness derived from inflammatory mediators and that TNF-*α* production may be due to inflammation rather than due to dryness per SE [30]. It was demonstrated that nourishing Yin and eye pill inhibit the inflammatory response of the ocular surface by interfering with the signaling molecules that are key to the P38MAPK signaling pathway by detecting the P38MAPK protein content in the rat conjunct skin [31]. The triggering factors of our experiment were different from the above study, but the results obtained were consistent.

Our experimental results showed that the expression of TNF-*α* and IL-1*β* in T2DM patients was significantly and negatively correlated with BUT and chimeric. The corneas of patients with various types and severity of T2DM homoeopathy were compared and observed in detail using laser confocal corneal microscopy, and it was found that the severity of disease in patients with T2DM homoeopathy correlated with the distribution of inflammatory cells in the corneal epithelium and whether they were activated or not [32]. The values of Schemer II experiment were significantly reduced in patients with T2DM, while Schemer I experiment and BUT experiment values did not show significant differences compared to the comparison group [33]. This is inconsistent with the results of the present experiment, considering that Goebbels' experimental subjects were T2DM patients related. The experimental results showed that there was also a significant impregnation of inflammatory factors in milder than T2DM homoeopathy, suggesting that inflammation occurs early in T2DM homoeopathy, indicating that this unnoticeable inflammation predates the epithelial damage visible to the naked eye. Therefore, the inflammatory response may be a cause of epithelial damage [34]. Therefore, whether inflammation is the initiating factor could be a new starting point for further studies. Therefore, appropriate prophylaxis is given to T2DM patients at the beginning of T2DM homoeopathy, i.e., when epithelial damage has not occurred. The severity of T2DM homoeopathy varies, and their expression varies, reflecting the course of disease development, i.e., as the inflammatory response gradually increases, the extent of the conjunct area it affects becomes more extensive [35]. The experimental results showed that the expression of TNF-*α* and IL-1*β* in retinal cells of T2DM patients was negatively correlated with the values of BUT test and Schemer I test, so it is possible to initially assess the level of inflammation in the ocular surface based on clinical examination results, and it is crucial to select the timing of inflammatory treatment relatively accurately [36].

There are some deficiencies in this study. We found that the serums TNF-*α* and IL-1*β* in patients with T2DM homoeopathy were significantly higher than those in normal people, and the serums TNF-*α* and IL-1*β* in patients with T2DM homoeopathy were higher than those in ordinary diabetic patients. Serum levels of TNF-*α* and IL-1*β* were highest in patients with nonproliferation of T2DM homoeopathy. Correlation analysis showed that serums TNF-*α* and IL-1*β* were significantly positively correlated. The above all suggest that the two play a synergistic role in the occurrence and development of patients with T2DM homoeopathy, but the two belong to the upstream and downstream relationship or the same level relationship, which needs further discussion. In addition, we found that both serums TNF-*α* and IL-1*β* were related to body mass index and disease course, while T2DM homoeopathy patients had higher body weight, which further confirmed that serums TNF-*α* and IL-1*β* were significantly related to DR progression.

In summary, serums TNF-*α* and IL-1*β* are significantly elevated in patients with T2DM homoeopathy and gradually increase with disease progression. Combined detection of serums TNF-*α* and IL-1*β* can help determine the severity of the disease and assess prognosis. Our study is expected to provide a basis for preventing and diagnosing T2DM homoeopathy.

## Figures and Tables

**Table 1:** Comparison of baseline information of patients in the two groups.

Group	Average age (years)	Sex (M/F)	Body mass index (kg/m^2^)	Body weight (kg)
Comparison group (50)	60.90 ± 1.71	24/26	23.05 ± 2.23	54.35 ± 1.29
Observation group (50)	61.10 ± 1.62	23/27	23.40 ± 2.03	54.10 ± 1.31
*χ* ^2^/*t*	-0.600	0.040	-0.821	0.962
*P*	0.550	0.841	0.414	0.339

## Data Availability

No data were used to support this study.
